# Cell-Intrinsic Type I Interferon Signaling as a Pleiotropic Orchestrator of CD4 T Cell Immunity

**DOI:** 10.3390/biom16030374

**Published:** 2026-03-02

**Authors:** Eugene Baffoe, Adhithya Aaron Anand, K. Kai McKinstry

**Affiliations:** Immunity and Pathogenesis Division, Burnett School of Biomedical Sciences, College of Medicine, University of Central Florida, Orlando, FL 32827, USA; eugene.baffoe@ucf.edu (E.B.); adhithya.anand@ucf.edu (A.A.A.)

**Keywords:** type I IFN, CD4 T cell, Th subsets, memory, STAT1, STAT4

## Abstract

Type I interferons (IFN-I) are pleiotropic cytokines best known for their antiviral impacts. However, they are known to also impact immune responses outside of viral infection through directly signaling many populations of innate and adaptive immune cells. Here, we focus on the complex body of findings from viral, bacterial, and parasitic infection models, cancer and autoimmunity studies, as well as in vitro experiments using human and murine T cells, demonstrating that IFN-I can be directly sensed by CD4 T cells. Such signaling has been shown to influence many central aspects of antigen-specific CD4 T cell responses, including proliferation, apoptosis, effector subset differentiation, and memory formation. These effects are frequently divergent and sometimes opposing, likely reflecting how differences in variables related to the IFN-I signal, overall inflammatory milieu, and the CD4 T cell integrate to shape outcomes. Indeed, we discuss findings supporting a framework in which dynamic engagement of canonical and non-canonical signaling pathways downstream of IFN-I, which are contingent on a cell’s activation state, play a key role in determining whether and how IFN-I promotes, restrains, or otherwise reprograms CD4 T cell fates. Together, these observations highlight the impressive scope of regulation that IFN-I signals to CD4 T cells can exert, parallel to its actions on other immune and non-immune cell types. They also suggest that harnessing such signaling could offer powerful therapeutic strategies to shape CD4 T cell immunity in diverse context-dependent situations.

## 1. Introduction

**Figure 1:**
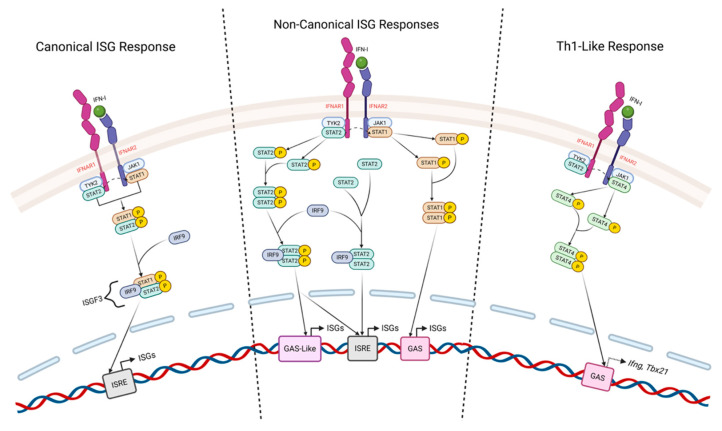
Canonical and non-canonical IFN-I signaling pathways in T cells. IFN-I signals through the heterodimeric IFNAR receptor (IFNAR1/IFNAR2), activating JAK1 and TYK2 and promoting phosphorylation of STAT proteins that regulate transcriptional programs. ‘Canonical’ IFN-I signaling induces the phosphorylation of STAT1 and STAT2, which associate with IRF9 to form ISGF3 complexes that translocate to the nucleus to drive expression of interferon-stimulated genes (ISGs) through interferon-stimulated response elements (ISREs) (**left panel**). ‘Non-canonical’ IFN-I signaling encompasses alternative STAT assemblies, including STAT2–IRF9 complexes, STAT2 homodimers, and STAT1 homodimers, enabling ISG expression through ISREs, GAS, and GAS-like regulatory elements (**central panel**). IFN-I can also promote Th1-like transcriptional programs through STAT4 activation, leading to GAS-dependent gene expression, including induction of *Ifng* and *Tbx21* (T-bet) (**right panel**). Dashed boundaries delineate distinct signaling modes, highlighting how IFN-I can engage multiple STAT-dependent pathways to elicit diverse transcriptional outcomes in T cells. Created in Biorender. Anand, A. (2026) https://BioRender.com/y2syq4j (accessed on 23 February 2026).

Since their discovery in the late 1950s [1], a considerable amount of work has focused on understanding the regulation and impacts of type I Interferons (IFN-I) in varied settings of health and disease. The best characterized IFN-I family members consist of IFNβ and multiple IFNα isoforms that all signal through a single heterodimeric receptor commonly referred to as the IFN-α/β receptor (IFNAR), which is composed of the IFNAR1 and IFNAR2 subunits (Figure 1). Other less studied members of the IFN-I family include IFNδ, IFNε, IFNκ, and IFNω [2]. Virtually all cell types can express IFNAR, and a remarkably broad range of cells can produce IFN-Is in response to several different molecular triggers [3,4]. IFN-Is are exceptionally pleiotropic cytokines, but they are best known for their roles in orchestrating intracellular protection against viruses. This is achieved primarily through the induction of hundreds of interferon-stimulated genes (ISGs) that mediate diverse activities, including the inhibition of protein synthesis, the degradation of viral RNA, and the restriction of viral attachment, membrane fusion, and endocytosis. IFN-Is are also potent regulators of inflammation through directly impacting the responses of many subsets of innate immune cell types, with the potential to drive both positive and negative outcomes [5]. More recent studies have shown IFN-Is to also have broad and varied impacts in regulating inflammation in the settings of cancer [6], autoimmunity [7,8], and bacterial infection [9], demonstrating that their influence extends far beyond defense against viral pathogens.

Given the remarkable scope of regulation mediated by IFN-Is, it is unsurprising that they have also been found to profoundly impact adaptive immune responses in a variety of settings, both positively and negatively. Defining how IFN-I signals regulate the response potentials of individual cell types is thus an important goal for understanding and harnessing their immunomodulatory potential. Here, we focus on the growing body of literature identifying IFN-Is as potent modulators of a diverse array of CD4 T cell attributes. IFN-Is have strong *indirect* influences on T cell responses through their impacts on other cell types that interact with T cells, most notably antigen-presenting cells (APC) and B cells, and through shaping inflammatory cytokine milieus in which they are activated and respond. These impacts can be critical in determining several aspects of CD4 T cell effector differentiation and memory formation and thus are important in determining the outcomes of immune responses. Often overlooked, though, are the impacts of IFN-I signals that are *directly* received through binding IFNAR on the surface of CD4 T cells. Understanding how such signaling can regulate CD4 T cell potentials is important for providing insight into the causality of outcomes involving complex immune circuits, especially given the central role of CD4 T cells as ‘helpers’ of other adaptive immune cells, regulators of innate immune cell activation, and potent effector cells in their own right. We will thus focus our discussion largely to findings demonstrating the surprisingly broad scope, and sometimes contradictory impacts, of IFN-I signals received by CD4 T cells. We will also discuss evidence suggesting that dynamic changes associated with T cell activation status play a key role in dictating how intrinsic IFN-I signaling can differentially impact their response capacity. Together, this body of investigation highlights the potential for properly timed and targeted IFN-I signals to be exploited as a tool to optimize various aspects of CD4 T cell effector responses and memory generation across many different therapeutic and vaccine settings.

## 2. Basic Elements of Intracellular IFN-I Signaling

A number of excellent review articles have discussed in detail the subtleties of intracellular IFN-I signaling [5,10], as well as the many molecular triggers able to initiate, promote, and regulate IFN-I production [11,12]. Here, we will provide only a concise summary necessary to better appreciate key aspects of IFN-I signaling as it relates to findings that we will discuss further on, focusing on CD4 T cell responses (Figure 1). IFN-I signals downstream of binding IFNAR are mediated primarily through ‘canonical’ and ‘non-canonical’ Janus kinase (JAK)—Signal transducer and activator of transcription (STAT) pathways. More recent studies have also identified STAT-independent ‘non-classical’ IFN-I signaling modes that are mediated through MAP-kinase and PI3K/mTOR pathways, as well as other signaling cascades [13].

Classically, upon IFN-I binding to IFNAR, JAK1 and tyrosine kinase 2 (TYK2) are activated, which in turn leads to the downstream phosphorylation of the transcription factors STAT1 and STAT2. Phosphorylated STAT1 and STAT2 form heterodimers that can associate with Interferon regulatory factor 9 (IRF9) to form the Interferon-stimulated Gene Factor 3 (ISGF3) complex. ISGF3 then moves to the nucleus, where it can bind to interferon-stimulated response elements (ISREs) in gene promoters, which in turn activate the transcription of a broad array of ISGs [14,15,16].

Notably, activated STAT1 and STAT2 are not limited to serving as constituents of the ISGF3 complex; each can form homodimers that are able to independently drive non-canonical IFN-I signaling pathways. STAT1 homodimers, sometimes called gamma-activated factor (GAF), bind to gamma-activated sequence (GAS) elements and regulate distinct gene subsets that are involved in driving pro-inflammatory responses more commonly associated with IFNγ regulation [3,14,17]. STAT2 homodimers, on the other hand, lack a strong DNA-binding domain, but can bind IRF9 to form ISGF3-like complexes. These STAT2/IRF9 complexes exhibit altered kinetics and gene usage compared to canonical ISGF3 [14,16,18,19]. STAT1/IRF9 complexes can also form, but their functional significance is not as clearly defined [16,19].

Beyond these pathways, IFN-I signaling can engage other STATs in context-dependent settings [20,21]. These include STAT4, the expression of which is restricted largely to immune cells [22], and is most commonly associated as a critical transcription factor needed for the full polarization of Th1 CD4 T cells. STAT3 can also be activated in mouse and human T cells by IFN-I signaling [23]. IFN-I-activated STAT3 has been shown to have diverse impacts in CD4 T cells. For example, it can downregulate STAT1 activation and downstream gene expression [24] and, in STAT1-deficient T cells it has been shown to contribute to IFN-I-induced proliferation and survival in some [25] but not other [26] studies. CD4 T cells can also activate STAT5 in response to IFN-I signaling, which can contribute to productive rather than suppressive signaling [27]. Observations also support that STAT6, commonly associated with Th2 differentiation of CD4 T cells, can be activated downstream of IFN-I signaling in T cells [28]. Outcomes of context-dependent intrinsic IFN-I signaling in T cells may thus reflect an integration of canonical, non-canonical, and non-classical (STAT-independent) pathways.

## 3. IFN-I’s Impacts Through the Th1/Th2 Prism

In line with its well-known antiproliferative impacts on other cell types, several studies in the 1980s found a potent inhibitory impact of recombinant IFN-I on mitogen-driven T cell responses when added to cultures of human peripheral blood mononuclear cells (PBMC) [29,30,31] vs. mixed results from earlier work using ‘purified’ IFN-I preparations, which in some cases may have contained contaminants, like growth factors, able to stimulate cells independently of IFN-I-driven impacts. Later studies in the early 1990s found that addition of IFN-I promoted Th1-like CD4 T cell differentiation, highlighted by strong IFNγ production, as well as cytotoxic potential, in cultures of human PBMC [32].

The first study using well-purified T cells to show a role for IFN-I in driving a Th1-like response from CD4, but not CD8 T cells, was published in 1993, also using human T cells [33]. Importantly, this work leveraged plate-bound anti-CD3 antibodies for T cell stimulation rather than APC, thereby restricting the impact of IFN-I to direct signals received by the activated T cells. Similar findings were made using T cells obtained from neonatal donors [34] that also identified upregulation of the immunosuppressive cytokine IL-10 by IFN-I stimulation, a finding also made by others in analyses of CD4 T cells isolated from adult PBMC which we will discuss in Section 6.2 [35,36]. The latter study also identified IFN-I’s ability to restrict production of the Th2-associated cytokine IL-5 when added to T cell cultures. Combined, this early work thus solidified IFN-I’s pro-Th1, anti-Th2 activity. The ability of IFN-I signaling to T cells to restrict Th2 cytokine production was confirmed by later observations [37], including a direct suppressive effect of IFN-I signaling on CD4 T cell expression of the Th2 ‘master regulator’ transcription factor, GATA-3 [38].

Notably, the ability of IFN-I to promote Th1 characteristics was found to be demonstrably less potent compared to that of the prototypical Th1 driver, IL-12, when increasingly pure populations of naive human CD4 T cells were tested [39]. Studies assessing CD4 T cell clones generated in the presence of poly I:C, a strong promoter of IFN-I production, found maximal Th1 differentiation in the presence of IFN-I and IL-12, suggesting the potential for synergy between these cytokine signals. Furthermore, IFN-I can upregulate the expression of the receptor for IL-18 on activated T cells [40]. This offers an additional mechanism by which IFN-I can promote Th1 development, as IL-18 can synergize with IL-12 to potentiate Th1 functionality.

Mice deficient for the IFN-I receptor (IFNAR1) were first described in 1994 [41,42], which, combined with other technological advances, has allowed for a detailed assessment of IFN-I’s potential to impact antigen-specific T cell responses through intrinsic signaling in a broad array of in vivo models. Combined with an improved resolution of activation states beyond the Th1/Th2 dichotomy, mouse studies have complemented clinical analysis in developing a more complete understanding of how IFN-I signals to CD4 T cells can modulate their functions and impact disease outcomes, as we will discuss in the following sections.

## 4. Of Mice and Men

As compared to the impacts of other key factors controlling Th1/Th2 fates, like IFNγ, IL-12, and IL-4, which were shown to operate similarly in reductionist models using human or mouse T cells, the findings discussed in Section 3 indicating roles for direct IFN-I in promoting Th1-like responses were almost exclusively made using human cells. Using naive T cells obtained from T cell receptor (TcR) transgenic mice, in vitro stimulation with cognate peptide presented by irradiated syngeneic APCs revealed that type I interferon (IFN-I) failed to induce IFNγ production, in contrast to the addition of the canonical Th1-polarizing cytokine, IL-12 [43]. However, the addition of IFN-I in combination with IL-12 to naive CD4 T cell cultures was shown to inhibit Th2 development, as judged by IL-4 production [43], though other head-to-head experiments also found robust anti-Th2 impacts only with human CD4 T cells [38]. Later work provided molecular explanations for the divergent impacts of IFN-I between species. First, IFN-I was shown to activate STAT4 in cultured human cells [44], but not (or only very weakly [45]) in mouse T cells [46]. As experiments with knockout mice had recently defined a requirement for STAT4 in generating IL-12-dependent Th1 cells [47,48], the inability of murine STAT4 to be effectively engaged by IFN-I signaling thus provided mechanistic clarity to the outcomes summarized above. Further analysis on the impacts of IFN-I on Th1 development in human vs. murine CD4 T cells identified a mutation in the mouse STAT2 protein preventing it from recruiting STAT4 to the IFNAR complex [49]. Unexpectedly, though, experiments with mice expressing a *STAT2* gene ‘fixing’ this mutation found that IFN-I still could not support robust Th1 development [50], indicating that other differences in the regulation of IFN-I signaling also impact outcomes. Indeed, a second attribute distinguishing IFN-I signaling in human vs. mouse T cells is that STAT4 can directly interact with the human IFNAR2 subunit of IFNAR, but cannot with murine IFNAR2 [51]. Further experiments suggested a third potential mechanism underlying the more potent ability of IL-12 vs. IFN-I to drive Th1 development: while IL-12 stimulation drives the sustained activation of STAT4, leading to stable expression of the Th1 ‘master regulator’ transcription factor T-bet, IFN-I signaling only transiently activates STAT4 [52], which does not allow stable T-bet expression over time as compared to the activity of IL-12 [53]. Finally, while IL-12 signaling results in upregulation of the IL-12 receptor, helping to potentiate Th1 differentiation by making cells more sensitive to IL-12, IFN-I receipt downregulates IFNAR expression, thus limiting its potential for the sustained signaling needed to promote Th1 identity [54]. IFN-I thus appears to be subordinate to other lineage-defining cytokine signals in promoting Th1 differentiation. Nevertheless, at least in some situations, IFN-I can promote strong Th1 hallmarks, even in murine T cells, as we will discuss in the next section.

## 5. A Tale of Two STATs

In contrast to conclusions drawn from earlier research, seminal in vivo studies by Biron and colleagues in 1999 found that IFN-I signals could support the development of robust antiviral CD8 T cell responses, defined by strong cytotoxicity and IFNγ production (often termed ‘Tc1’), against Lymphocytic Choriomeningitis Virus (LCMV) in mice deficient for IL-12 [55]. In the same infection model, it was shown that STAT1 expression by CD8 T cells inhibited this outcome, and in a reductionist model, that the addition of IFN-I to cultures of STAT1-deficient, but not wildtype, CD8 T cells stimulated with anti-CD3 antibody drove robust IFNγ production [56]. These observations thus defined a central negative role for STAT1 in determining the outcome of IFN-I signaling in T cells, and revealed that, at least in the absence of STAT1, IFN-I signaling can have potent pro-Th1-like activities in murine T cells. Indeed, just as discussed above for human T cells, STAT4 expression was shown by Biron’s group to be required for the development of Tc1 responses driven by IFN-I in mouse CD8 T cells using STAT4-deficient mice and other approaches [57]. Furthermore, in a model where IFN-I was shown to exert direct inhibitory effects on Th1 cells, the treatment correlated with increased STAT1 activation and reduced activated STAT4 levels [58], consistent with earlier in vitro findings (a potential basis for divergent effects of IFN-I in potentiating vs. inhibiting T cell responses is discussed in Section 8).

Does STAT1 expression also limit the ability of IFN-I to drive Th1 differentiation in CD4 T cells? We recently asked this using in vitro cultures of naive WT or STAT1-deficient mouse CD4 T cells stimulated with APCs and cognate peptide. We found that the addition of IFN-I to such cultures did not promote Th1 attributes in WT CD4 T cells, but did upregulate T-bet, as well as its paralog, Eomesodermin (Eomes), and promoted robust IFNγ production in ~80% of STAT1-deficient cells [59]. In other work, we found Eomes to mediate strong Th1 differentiation even in the absence of T-bet [60], showing the potential for IFN-I to promote Th1 imprints independently through both T-bet and Eomes induction. Furthermore, we found that the pro-Th1 impact of IFN-I in the STAT1-deficient CD4 T cells correlated with STAT4 activation [59]. These observations thus mirror the findings made with CD8 T cells discussed above, indicating similar governance over IFN-I signaling by STAT1 and STAT4 in both T cell subsets. There are additional observations supporting a pro-Th1 role for IFN-I signals received by murine CD4 T cells. For example, in a *Listeria monocytogenes* challenge model, bacteria-specific IFNAR-deficient CD4 T cells responding in WT host mice expressed less IFNγ compared to WT CD4 T cells recognizing the same epitope [61]. Interestingly, research using CD4 T cells isolated from younger and older donors found that older cells do not activate STAT1 as efficiently as young cells after IFN-I signaling [62]. Whether such differences in STAT1 activation mark older T cells responding in vivo, and whether this regulation may have impacts on the Th1 response potentials of old vs. young T cells responding in environments where IFN-I is present, requires further study.

In line with the observations discussed above for conventional CD4 and CD8 T cells, the impacts of IFN-I in maximizing IFNγ production and cytotoxic functions by human mucosal-associated invariant T (MAIT) cells have been shown to be STAT4-dependent [63], though similar IFN-I-dependent impacts in murine MAIT cells in a model of bacterial pneumonia were correlated with STAT1 activation (it is important, though, to point out that STAT4 activation was not addressed in this study) [64]. STAT4-dependent IFNγ production in response to IFN-I stimulation has also been shown for γδ T cells, suggesting conserved intracellular STAT-dependent signaling requirements for IFN-I-dependent Th1-like functionality across diverse T cell populations [65].

## 6. IFN-I’s Impacts on CD4 T Cells Beyond the Th1/Th2 Dichotomy

**Figure 2:**
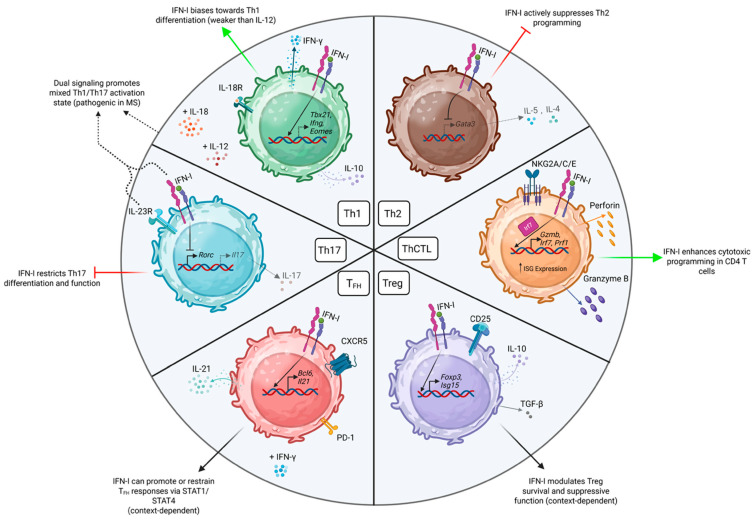
A summary of the impacts of IFN-I on major CD4 T cell effector subsets discussed in the text. Created in Biorender. Anand, A. (2026) https://BioRender.com/gy96zzf (accessed on 23 February 2026).

It is now clear that several other specialized subsets of CD4 T cells beyond Th1 and Th2 effectors contribute to protective and detrimental immune responses. Remarkably, IFN-I appears to have the capacity to impact the development of all major CD4 T cell subsets that have been described (Figure 2), including lesser-studied cells such as Th9 effectors [66]. Interestingly, just as for Th1 differentiation, conflicting roles for IFN-I signaling have been reported in the development and regulation of some of these effector subsets. Below, we summarize experimental findings that outline key aspects of this regulation for specific CD4 T cell effector types. In doing so, we will also highlight the wide array of clinical and experimental settings in which direct IFN-I signaling has been found to act to shape CD4 T cell responses.

### 6.1. Th17 Cells

Observations of stronger Th17 responses during *Listeria monocytogenes* infection in mice deficient for IFNAR and IL-12, with reciprocal decreases in IFNγ^+^ CD4 T cells, suggested that IFN-I can contribute to preventing the differentiation of Th17 cells. But this study used whole knockout mice and thus could not isolate the impacts of direct IFN-I signals to T cells in mediating these outcomes [61]. Our own observations comparing wildtype vs. STAT1-deficient CD4 T cells responding in WT hosts during Influenza A virus (IAV) infection, however, support this relationship. We found that the STAT1-deficient effectors express much-reduced levels of T-bet compared to WT cells, but we were surprised that the STAT1-deficient cells did not generate a Th17 response [59], as our previous studies found T-bet^−/−^ CD4 T cells to generate robust and highly protective Th17 responses in WT mice infected with IAV [67]. However, when we blocked IFN-I signaling during IAV infection in WT host mice by administering anti-IFNAR antibodies, a strong Th17 response was generated from the STAT1^−/−^ donor cells [59]. The impact of IFN-I to restrict Th17 production was most likely through direct signaling to the STAT1^−/−^ cells, as STAT1^−/−^ T cells responding to IAV in IFNAR-deficient mice also did not generate Th17 responses (unpublished observations). Similar impacts of IFN-I in restricting the development of Th17 responses have been seen in *Bordetella pertussis* infection in mice [68], in the mouse model of multiple sclerosis, Experimental Autoimmune Encephalomyelitis (EAE) [69], and also in studies assessing Th17 cytokine production by γδ T cells in mice infected with a *Francisella tularensis* subspecies [70]. Interestingly, while our IAV studies found IFN-I to inhibit Th17 programming in STAT1^−/−^ cells, other studies have found the anti-Th17 impact of IFN-I signaling to be STAT1-dependent [71,72]. This suggests that IFN-I may inhibit Th17 differentiation through both STAT1-dependent and -independent pathways.

Beyond suppressing Th17 polarization, in vitro experiments have found that IFN-I treatment can restrict Th17 cytokine production from already polarized Th17 cells [73]. In this study, the IFN-I treatment also boosted IL-10 production by the Th17 cells, which was implicated in downregulating Th17 functionality. Similar results of increased IL-10 and decreased IL-17 were seen in human CD4 T cells treated with IFN-I [72], and mouse CD4 T cells treated with a STING agonist [74]. The authors showed that the suppressive impact of STING signaling on Th17 development did not require autocrine/paracrine IL-10 signaling by using IL-10-deficient CD4 T cells. In contrast, our own results analyzing WT and IL-10-deficient CD4 T cells responding to IAV in WT mice did show a clear inhibitory impact of CD4 T cell-derived IL-10 on the generation of anti-viral Th17 responses [75]. Thus, IFN-I signaling may drive both IL-10-dependent and independent modes of Th17 suppression. Finally, data indicate that in the presence of IL-23, IFN-I can help to promote a mixed Th1/Th17 activation state that has been found to be highly pathogenic in a number of settings, including multiple sclerosis [76].

### 6.2. Regulatory T Cells (Tregs)

FoxP3^+^ regulatory CD4 T cells (Tregs) are a critical element of immune responses that have been found to be impacted by IFN-I signaling. In a colitis model, IFN-I was shown to be important in maintaining suppressive Treg impacts in vivo by comparing IFNAR-sufficient or -deficient Tregs transerred to *Rag1*-deficient mice that were also given disease-driving CD45RB^high^ WT CD4 T cells [77]. IFN-I signals received by Tregs have been reported to be important in reducing disease severity in EAE studies [78]. Similar outcomes have been seen in mouse models that identified a role for IFN-I in contributing to their regulatory capacity in select contexts [79]. More reductionist in vitro experiments focused on induced Tregs found IFN-I signals to enhance their survival and suppressive functions, but to impair their proliferation [80]. Consistent with these relationships, harnessing the ability of IFN-I to promote Treg responses in order to dampen damaging immune responses has been explored in mouse models of rheumatoid arthritis [81], and allogeneic heart transplantation [82]. Conversely, blockade of IFN-I signals to Tregs has been shown to help unleash immune responses and improve outcomes in cancer models [83].

In contrast to the observations described above, there are also findings suggesting that IFN-I signals can negatively impact Treg-mediated regulation. For example, in studies of obese mice, IFN-I derived from plasmacytoid DC was shown to deplete Tregs in adipose tissues, leading to increased chronic inflammation [84]. A negative role for direct IFN-I signals on Tregs was also seen during acute LCMV infection, with mice harboring Treg-specific deletion of IFNAR displaying enhanced Treg activity, impaired antiviral T cell responses, and worsened outcomes [85], with similar outcomes seen in studies of chronic LCMV infection [86]. Thus, the impacts of IFN-I signals to Tregs appear to be context-dependent in terms of their impact. Interestingly, expression of the hallmark ISG, ISG15, may be critical to protect Tregs from excessive IFN-I signaling to help preserve a level of Treg function in the face of strong inflammatory conditions [87]. In contrast, ISG15 has been shown to promote IFNγ production by human [88] and mouse T cells [89] in some settings. These results suggest complex regulation of IFN-I feedback loops that may either potentiate stimulatory or inhibitory impacts depending on Th subset identity. Indeed, targeting ISG15 [90], as well as other critical ISGs involved in IFN-I feedback regulation, may offer novel strategies to fine-tune T cell responses.

While IFN-I signals in vitro have been shown to promote production of IL-10 by Tregs, IL-10 can also be produced by specialized FoxP3^−^ CD4 T cell effectors, often termed ‘Tr1’ cells. More potent IL-10 production was seen from human FoxP3^−^ CD4 T cells activated in vitro in the presence of IFN-I [91], with IFN-I and IL-10 itself found to differentiate Tr1 from naive human CD4 T cells [92]. The ability of IFN-I to promote IL-10 production in at least some settings appears to be independent of STAT1 and STAT4, instead relying on STAT2 and STAT3 as revealed by studies using human CD4 T cells [93]. These results highlight the diverse biological outcomes possible downstream of the complex integration of IFN-I signals through the distinct STAT pathways introduced earlier. During IAV infection, production of IL-10 driven by direct IFN-I signaling to T cells requires IRF4 and Blimp-1 [94], further supporting findings that IFN-I signaling can engage multiple downstream signaling pathways in T cells.

### 6.3. Follicular Helper Cells (T_FH_)

Follicular helper cells (T_FH_) are critical for optimal antibody responses and for the development of long-lived humoral immunity. Early in vivo studies found that IFN-I signaling to DC after protein immunization was critical for the optimal generation of T_FH_, and that IFN-I might impact T_FH_ through indirect impacts on B cells [95]. However, direct IFN-I signals to CD4 T cells were shown in vitro to act through STAT1 to induce many T_FH_ hallmarks, including the T_FH_ ‘master regulator’ transcription factor, Bcl6, the chemokine receptor CXCR5, as well as PD-1 (a molecule often used as a T_FH_ marker), but not the capacity to produce the T_FH_-linked cytokine, IL-21 [96]. Interestingly, STAT4 activation downstream of IFN-I signals received by CD4 T cells has been shown to promote IL-21 production by T_FH_ in a mouse Lupus model, with similar regulation found analyzing T_FH_ from Lupus patients [97]. IFN-I signals received by T_FH_ were also shown to protect them against NK cell-mediated depletion in separate Lupus studies [98]. These observations indicate that, at least in some situations, STAT1 and STAT4 activation mediated by IFN-I may act in synergy to promote T_FH_ responses. IFN-I signals have also been shown to help promote the development of extrafollicular (CXCR5^−^) IL-21-producing CD4 helper T cells in vitro [99,100], suggesting a separate layer of control over T cell-dependent humoral immunity that can be impacted by IFN-I.

In contrast to the work summarized above, a separate body of experiments has defined an inhibitory role for IFN-I in the generation of T_FH_. For example, virus-specific CD4 T cells lacking IFNAR transferred into mice infected with LCMV developed a stronger T_FH_ response compared to wild-type CD4 T cells [101]. Furthermore, in a malaria infection model, T_FH_ responses were found to be restricted by IFN-I signals that operated not by impairing T_FH_ differentiation, but by directly promoting T-bet^+^Blimp1^+^ effector CD4 T cells able to constrain T_FH_ responses through the production of IFNγ and IL-10 [102]. Taken together, it is thus likely that the impacts of IFN-I signals received by CD4 T cells on generating T_FH_ are nuanced and themselves impacted by other variables in the inflammatory environment.

### 6.4. Cytotoxic CD4 T Cells (ThCTL)

Early studies examining the impacts of IFN-I signaling on CD8 T cells found it to promote increased cytotoxic potential. More recent studies have identified cytotoxic CD4 T cells as a distinct subset, sometimes referred to as ThCTL, able to impact outcomes of several different immune responses through a number of distinct cytotoxic mechanisms [103]. Using single-cell genomics paired with functional studies, IFN-I signaling in CD4 T cells has been found to upregulate several cytotoxic markers, with CD4 T cells bearing cytotoxic hallmarks as well as a strong ISG signature identified in autoimmune patients [104]. Mechanistically, maximal impacts of IFN-I on promoting cytotoxic differentiation were shown to require expression of the transcription factor IRF7, itself an ISG. Other studies have identified a role for direct IFN-I signals to CD4 T cells for maximizing their cytolytic potential in the lungs during IAV infection through a STAT2-dependent pathway that also served to promote maximal T-bet expression [105]. Our own studies have shown that effective ThCTL induced by IAV infection can develop in the absence of T-bet expression by CD4 T cells [60], although the T-bet-deficient cells expressed lower levels of granzyme B, a marker used to define ThCTL in most settings. This is consistent with the concept that multiple transcriptional pathways able to be impacted by direct IFN-I signals may support similar levels of cytotoxic potential in CD4 T cells. Indeed, an IFN-I-dependent, STAT3-mediated pathway has been shown to improve cytotoxic functions in CD8 T cells responding to tumor challenge [106]. Likewise, direct STAT1-dependent IFN-I signaling to memory CD8 T cells was shown to enhance granzyme B expression in both mouse and human cells [107]. Whether and how these signaling pathways downstream of IFN-I receipt can integrate to impact ThCTL development and/or function will require further study.

### 6.5. Th_ISG_?

Technologies, including single-cell RNA sequencing (scRNAseq), have revealed a depth of heterogeneity within T cell populations responding in vivo that is not easily appreciable by other approaches. An unexpected and almost universal finding within single-cell RNAseq data sets focused on T cells responding in a wide array of settings has been well-defined subsets of cells marked by strong expression of ISGs [108] (with many more examples having been published in the past few years that will not be cited here). This suggests that T cells with differing strengths of IFN-I-dependent programming (often concomitant with hallmarks of other Th/Tc subsets) are routinely generated as part of immune responses, which in turn suggests that such cells may play underappreciated roles. Further studies are required to determine whether such T cells constitute stable effector and/or memory subsets (Th_ISG_) vs. transitional populations, and how such cells relate to better characterized (archetypical) populations. Another critical goal will be to determine the positive vs. negative impacts of ISG-marked T cells in context-dependent situations, as well as the regulation underlying their development, especially given the potential complex nature of IFN-I-dependent signaling through canonical and non-canonical pathways discussed above. In the long-term, these avenues of research may reveal novel ways in which IFN-I signals can be targeted to CD4 T cells to improve outcomes in a variety of disease settings.

## 7. Life and Fate

Beyond influencing effector subset differentiation, another critical way in which IFN-I signals impact T cell responses is through regulating the survival of activated cells, although widely varying outcomes have been reported. For example, IFN-I signaling can have direct antiproliferative [109,110] and pro-apoptotic [111] effects on T cells. Such outcomes can be driven by IFN-I-dependent increases in expression of the death receptor, FAS, and its ligand [112], while other studies have identified IFN-I-dependent upregulation of several inhibitory surface molecules on T cells, including PD-1, BTLA, and LAG-3 [113,114,115,116]. While increased expression of these molecules is consistent with regulatory or exhaustion-associated transcriptional programs, it is important to note that their functional consequences vary across experimental systems. In many contexts, inhibitory receptor induction likely reflects part of a broader IFN-I–induced regulatory circuit whose net impact depends on concurrent signals and disease state. For human CD4 T cells, IFN-I stimulation in vitro has been found to directly upregulate expression of pro-apoptotic molecules [117], and sustained IFN-I signals to CD8 T cells have been found to trigger complex metabolic changes, making them more susceptible to apoptosis [118] and to activation-induced cell death upon TcR restimulation [119]. The pro-apoptotic impacts of type I signaling have been linked to STAT2-dependent signaling [120]. There is also evidence of pro-apoptotic impacts of IFN-I during viral infection on ‘bystander’ CD8 T cells that are not specific for viral antigens [121]. Interestingly, IFN-I can even promote the death of CD4^+^ thymocytes in fetal thymic organ cultures infected with HIV [122].

In contrast to the results summarized above, a separate body of findings describes pro-survival and proliferative impacts of IFN-I for CD4 T cells [123,124], with similar impacts found for CD8 T cells [125,126]. STAT1 activation appears to be important for at least some aspects of the anti-apoptotic impacts of IFN-I signaling; for example, by upregulating Bcl-x expression [124]. In a murine model, enhanced IFN-I signaling was shown to promote the survival of CD8 T cells within tumors, correlating with enhanced Bcl-x expression, and to improve the protective efficacy of adoptively transferred tumor-specific CD8 T cells [127]. In this study, enhanced IFN-I signaling to CD8 T cells was achieved by increasing surface expression of IFNAR by interfering with its ubiquitination and subsequent degradation, offering a potentially useful strategy to explore the impacts of increasing the efficiency of cell-specific IFN-I signaling in other settings as well, with fewer off-target impacts than are associated with approaches using exogenous IFN-I treatment. Another way by which IFN-I can protect T cells from death and promote clonal expansion in vivo is through protecting the cells from NK-mediated killing by upregulating molecules on the T cell surface, like MHC-I, that provide inhibitory signals to NK cells [128,129]. Recent studies have found that the route of vaccination can have a profound impact on how IFN-I is received by T cells, which impacts their response potential—either suppressing (subcutaneous) or enhancing (intravenous) responses [130]. A complicating aspect of interrogating how IFN-I impacts T cell expansion is that experimental contexts seem to matter more than for the investigation of some other cytokines [131], likely due to wide differences in its relative induction by different pathogens, or other disease-driving triggers. For example, while direct IFN-I signals are critical for optimal expansion of virus-specific CD8 T cells during LCMV infection, the same pattern was not found by the same authors in a model of vaccinia virus infection expressing the same LCMV epitope [132].

## 8. A Wrinkle in Time

**Figure 3:**
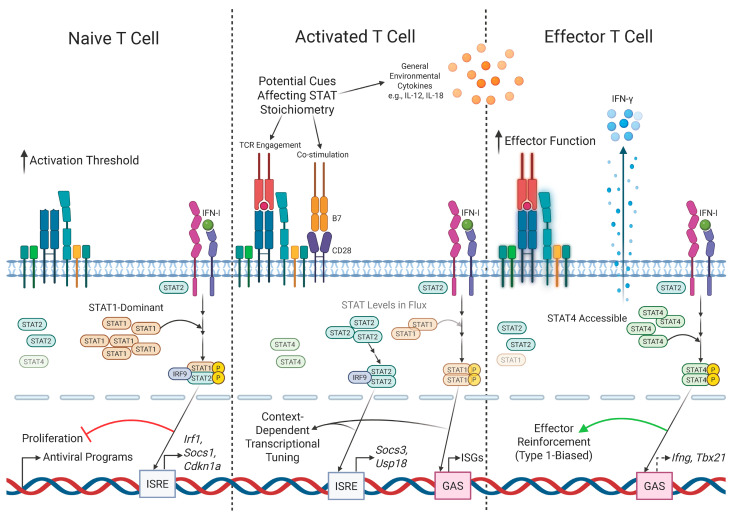
A model for context-dependent changes in IFN-I signaling dictated by T cell activation state. Dynamic changes in levels of STAT expression regulated by T cell activation state dictate outcomes of IFN-I signaling. In naive T cells, IFN-I signaling proceeds predominantly through STAT1-dependent pathways, favoring canonical ISGF3 formation and ISRE-driven ISG expression that is associated with antiproliferative and regulatory ISGs, including putative mediators such as Irf1, Socs1, and Cdkn1a (**left**). Upon activation, dynamic changes in STAT expression and stoichiometry occur, influenced by signals including environmental cytokines, receipt of costimulatory signals, and the strength of TcR stimulation. Thus, depending on the context of activation, IFN-I signaling may engage alternative STAT assemblies enabling both ISRE- and GAS-associated transcriptional outputs, such as Irf1 (a bridging ISG), Socs3, and Usp18 (**center**). In effector T cells, increased accessibility of STAT4 allows IFN-I signaling to directly promote Th1-like transcriptional programs through GAS elements, driving expression of Th1 hallmark genes like *Ifng* and *Tbx21*, with reduced impacts of STAT1-dependent IFN-I signaling (**right**). It is important to note that changes in other STAT-independent ‘non-classical’ signaling pathways may further complicate such temporal and quantitative changes in STAT expression to impact how IFN-I signals integrate with TcR and cytokine-derived cues to shape T cell fate and function. Created in Biorender. Anand, A. (2026) https://BioRender.com/b43egsl (accessed on 23 February 2026).

One explanation for the differences that have been reported in how IFN-I signals received by T cells impact their response potentials is linked to the kinetic changes in expression profiles of key downstream mediators of IFN-I signaling, particularly in the ratio of STAT1 and STAT4 (Figure 3). In a naive state, expression of STAT1 by CD8 T cells is relatively high, but after recognizing cognate antigen, STAT1 levels fall. This change in STAT1 expression, at least in part, underlies the reduced sensitivity to the antiproliferative effects of IFN-I signaling in activated vs. naive CD8 T cells [133]. Moreover, activated CD8 T cells also increase their expression of STAT4, with similar trends seen for CD4 T cells [134]. Elevated STAT4 levels in activated CD8 T cells are required for limiting STAT1-dependent IFN-I signaling, thus promoting CD8 T cell effector expansion [135]. Evidence for favored STAT4 vs. STAT1 signaling downstream of IFN-I has also been generated using activated human CD8 T cells, where STAT4-dependent signaling was shown to promote key effector functions like cytotoxicity [136], as discussed earlier. Thus, a major determinant of whether IFN-I signals result in predominantly suppressive vs. stimulatory signaling to T cells appears to relate to the timing of IFN-I receipt relative to their activation state, and the associated balance of STAT1 vs. STAT4 expression. One analysis of human CD8 T cells has, however, identified a pattern opposite to that discussed above—that IFN-I stimulation increased the proliferative capacity of naive cells while impairing responses of effector and memory cells [137]. It is possible that these disparate results relate to species-specific differences in IFN-I signaling potential discussed earlier.

There are no doubt other intracellular mechanisms that regulate how IFN-I signals shape outcomes of T cell responses, and these too might be impacted by activation status, or by other experimental variables. For example, changing levels of STAT3 expression in CD4 T cells relative to activation state may suppress canonical IFN-I signaling through several potential mechanisms [138], thus favoring STAT4-dependent pathways. Specific microRNAs expressed by CD8 T cells have also been found to be critical in restraining the suppressive impacts of IFN-I in vivo during viral challenge [139]. The extent to which patterns of STAT1/STAT4 expression and microRNA-dependent regulation of IFN-I signals in CD4 T cells mirror those in reported in CD8 T cells is not entirely clear.

Another body of evidence suggests that broader differences in T cell activation state act as a critical variable in determining how IFN-I signals can impact their response potential. Studies with human CD4 T cells in vitro, for example, found that IFN-I treatment had a suppressive impact upon TcR stimulation, whereas its addition to already activated CD4 T cells had no negative impacts [140]. At least one mechanism by which pretreatment with IFN-I has been found to restrict TcR-mediated T cell responses is by suppressing IL-2 production by CD4 T cells [141].

## 9. Remembrance of Things Past

Outcomes of direct IFN-I signaling to memory CD4 T cells also appear to be variable—either enhancing antigen-dependent proliferation and cytokine production or having negative impacts [142]. One potential explanation for such divergent findings is that certain subsets of memory CD4 T cells may be specialized to harness IFN-I as a stimulating signal. There is some evidence in support of this concept: IL-12 and IFN-I signals seem to promote distinct memory fates in CD8 T cells, with IFN-I preferentially instructing development of central vs. effector memory CD8 T cells, with opposite regulation by IL-12 [143]. Impacts on memory may also be dependent on the relative amount of IFN-I available, or other variables associated with the experimental models employed. For example, while a near-absolute dependence for direct IFN-I signals for memory CD8 T cell generation was found in a mouse model of acute LCMV infection [125], IFN-I does not seem to be important for establishing long-lived memory CD8 T cell memory capable of providing robust protection in mouse models of Sendai virus [144] or IAV infection [145]. In some cases, receipt of IFN-I by T cells may constitute one of many signals that could be harnessed to improve outcomes. For example, studies using vaccina virus infection found that early IFN-I or IL-12 signals to CD8 T cells during priming could support similar levels of memory development in mice [146]. Yet other studies have shown strong CD8 T cell memory responses to develop in the absence of both IFN-I and IL-12 that instead require CD27- and OX40-dependent co-stimulation [147]. The extent to which distinct pro-memory pathways can be harnessed to boost T cell immunity is not entirely clear, but there is evidence of synergy between CD27- and IFNAR-dependent signals to CD8 T cells, which can enhance their long-term survival [148].

Impacts of IFN-I on CD4 T cells have not been investigated as extensively, but it can be expected that it could also have multifaceted, situational impacts as described above for CD8 T cells in shaping memory. One plausible mechanism by which direct IFN-I may act, as has been shown for CD8 T cells, is by upregulating T-bet and thus favoring the generation of more short-lived effectors at the expense of effector cells with the capacity to form long-lived memory [149]. Indeed, we have shown improved memory generation (both circulating and lung tissue-resident) by CD4 T cells-deficient for T-bet compared to WT cells in the setting of IAV [67]. Impacts on CD4 T cell memory mediated by direct IFN-I signals may also involve the upregulation of Eomes: IFN-I can directly upregulate Eomes expression in CD8 T cells [150], and we have reviewed evidence supporting the capacity of Eomes to improve long-term CD4 T cell survival previously [151]. Whether other impacts of IFN-I signaling regulate CD4 T cell memory fitness, especially during non-Th1-biased responses, requires further investigation. Finally, that CD4 T cell memory can be impacted both by signals received by cells during their initial activation and at later timepoints during their effector response [152], leaves open the possibility that key IFN-I signals could act at either or both windows to regulate the efficiency of memory generation.

## 10. A Quiet Signal

A complicating factor in understanding how IFN-I impacts T cell responses in vivo is the delineation of its cellular sources, which are likely to be present both in secondary lymphoid organs, where T cells are first activated, and at peripheral sites, where effector T cells respond. While fibroblasts, epithelial cells, and many innate immune cells can produce IFN-I, plasmacytoid dendritic cells are often a chief source of IFN-I, especially during viral infection [153]. T cells can also produce IFN-I. Indeed, the capacity to produce IFN-I (IFNβ) by CD4 T cells has been found to contribute to the restriction of Th17 differentiation [154], and may, in other situations, provide a protective shield for effector CD4 T cells responding at sites of viral infection while also acting as an antiviral ‘effector’ cytokine [155]. IFN-I derived from CD4 T cells has also been found to contribute to optimal dendritic cell ‘licensing’ leading to improved CD8 T cell responses against tumors [156]. Interestingly, T-bet expression by CD4 T cells may play an important role in constraining autocrine IFN-I production and signaling, which can otherwise impair functional Th1 responses [157]. CD8 T cells have also been shown to produce IFN-I upon TcR triggering at levels sufficient to suppress HIV replication in vitro [158]. These observations warrant further situational exploration of how T cell-derived IFN-I impacts outcomes of immune responses, and how it is regulated by Th subset polarization and other variables.

## 11. Open Questions

Much has been learned in the past few decades about how direct IFN-I signals can impact T cell responses during immune responses, but unifying frameworks for how IFN-I signal strength, duration, or the context in which signals are received, are able to promote the diversity of outcomes discussed here remain incomplete. In addition, low-level ‘tonic’ IFN-I signals in the absence of overt immune triggers can also impact STAT expression levels and may thus regulate the response potentials of naive T cells prior to their recognition of specific antigens and subsequent receipt of costimulatory and cytokine signals, including IFN-I itself [159,160]. Even earlier, IFN-I signaling to thymocytes has been shown to impact T cell development, with lasting marks imparted to the mature T cells that exit the thymus to populate the naive T cell pool [161,162]. How these IFN-I signals modulate antigen-specific T cell responses has yet to be fully delineated.

Another challenge in fully understanding the role of IFN-I in shaping CD4 T cell immunity is the many variables that can independently impact its production by different cell types. For example, sex-dependent regulation of IFN-I levels and increased IFN-I production induced by obesity are well documented [163]. Furthermore, age-dependent shifts in STAT1 vs. STAT3 signaling in response to IFN-I have been reported in CD4 T cells [164], and increases in IFN-I-dependent signatures appear to be a key constituent of ‘inflammaging’ [165], a process that has profound consequences for T cell response potentials. How such shifts impact CD4 T cell subset balance or memory dynamics in the range of contexts examined in this discussion remains to be determined. There are also many observations supporting that different subtypes of IFNα can have different impacts on T cell responses [166,167], with data also supporting the specialized roles of IFNα and IFNβ in at least some settings [168]: whether certain IFN-I signals can preferentially direct specific elements of CD4 T cell responses is an intriguing possibility.

Finally, despite considerable progress in defining downstream IFN-I–STAT signaling modules, the upstream determinants that dynamically shape STAT stoichiometry and signaling outcomes in T cells remain only partially delineated (see Figure 3). Cytokine receptor signaling involves competitive recruitment and phosphorylation of STAT family members, and the relative access of different STATs to activated receptors appears to influence phenotype switching and transcriptional output in a manner dependent on the extracellular milieu and intracellular STAT abundance ratios, a phenomenon supported by models of STAT competition under varying cytokine inputs [169]. Moreover, TcR engagement can modulate the responsiveness of T cells to cytokines by tuning cytokine-mediated pathways, implying direct feed-forward and feedback interactions between antigen-specific signals and STAT-dependent networks [170]. Post-translational regulators such as SOCS proteins, differentially expressed upon activation, provide another layer that can alter STAT activation kinetics and pathway crosstalk [171]. Finally, integration of metabolic and ancillary signaling inputs, for example, mTOR-STAT interactions, suggests that environmental and cell-intrinsic states may further modulate STAT activation, availability, and nuclear targeting, yet the precise contributions of these axes to context-dependent STAT stoichiometry in IFN-I signaling in T cells are not well established [172]. Together, these observations point to a complex network of regulatory factors controlling STAT balance downstream of IFNAR that requires deeper mechanistic investigation.

## 12. Future Applications and Therapeutic Opportunities

The degree of complexity in the regulation of IFN-I signaling, combined with its potential to affect a broad scope of disease states, promises vigorous investigation and exciting discoveries to progress mechanistic understanding. Such advances are required to develop therapeutic strategies to modulate its impact on CD4 T cells in vivo. The findings discussed here indicate that targeting intracellular IFN-I-mediated signaling networks has the potential to fine-tune several elements of CD4 T cell responses. Of note, properly targeting IFN-I to CD4 T cells could potentially be harnessed both to potentiate or restrain their response potentials, offering a versatile clinical intervention. Of course, given the ubiquitous expression of IFNAR, receipt of IFN-I by other innate, adaptive, and non-immune cell populations can exert indirect modes of regulation on CD4 T cell responses that in some cases could supersede or even contradict the impacts of IFN-I on CD4 T cells themselves. This introduces a major challenge in developing strategies able to restrict or promote IFN-I dependent regulation, specifically in CD4 T cells or in other cell types. Fortunately, exciting advances in targeted delivery systems to specific cell types in vivo offer promise to move this goal forward.

## 13. Conclusions

Collectively, the literature supports a model in which IFN-I signaling outcomes in CD4 T cells are not intrinsically stimulatory or suppressive but instead emerge from the integration of several quantitative and contextual variables. These include (i) signal strength and duration of IFN-I exposure, (ii) timing of IFN-I receipt relative to TcR engagement, (iii) activation-state-dependent shifts in STAT abundance and accessibility, (iv) cytokine milieu and competing STAT-activating signals (e.g., cytokines like IL-12, IL-6, IL-10 that may be present in inflammatory milieus), (v) IFN-I subtype-specific receptor engagement dynamics, and (vi) concurrent cell-extrinsic effects of IFN-I on antigen-presenting cells and other immune subsets. Framing IFN-I responses through this integrative lens may provide a more predictive foundation for understanding their impacts in disease settings and for developing therapeutic strategies to modulate several key aspects of CD4 T cell responses to improve human health.

## Data Availability

No new data were created or analyzed in this study. Data sharing is not applicable to this article.
